# Community Cardiac Arrest as a Challenge for Emergency Medical Services in Poland

**DOI:** 10.3390/ijerph192316205

**Published:** 2022-12-03

**Authors:** Przemysław Żuratyński, Daniel Ślęzak, Kamil Krzyżanowski, Marlena Robakowska, Grzegorz Ulenberg

**Affiliations:** 1 Division of Medical Rescue, Faculty of Health Sciences with the Institute of Maritime and Tropical Medicine, Medical University of Gdańsk, 80-210 Gdańsk, Poland; 2 Department of Anesthesiology and Intensive Care, Oncology Center—Prof. Łukaszczyk Memorial Hospital in Bydgoszcz, 85-796 Bydgoszcz, Poland; 3 Department of Emergency Medicine, Pomeranian Academy in Słupsk, 76-200 Słupsk, Poland; 4 Department of Public Health & Social Medicine, Faculty of Health Sciences with the Institute of Maritime and Tropical Medicine, Medical University of Gdańsk, 80-210 Gdańsk, Poland; 5 Department of Interventional Nursing, Faculty of Health Sciences, Collegium Medicum in Bydgoszcz, 85-821 Bydgoszcz, Poland

**Keywords:** resuscitation, emergency medical services, cardiovascular diseases, healthcare systems, security

## Abstract

The problem of cardiac arrest, particularly out-of-hospital cardiac arrest (OHCA), is the subject of continuous research. The aim of this study was to analyze the use of an automated external defibrillator (AED) during the resuscitation of an adult in public places in Poland between 2015 and 2020. A retrospective analysis of the selected documentation obtained from AED distributors, the medical records obtained from the emergency call center, and the emergency medical teams was conducted. During the analysis period, there were 100 cases of recorded and documented use of AEDs in OHCAs in public places. In 70% of the cases, defibrillation was performed with an AED. This result could be higher, but the study’s methodology and limited access to data only allowed for this result. In Poland, there are no legal acts on the registration of automatic external defibrillators and their implementation. Appropriate registries should be introduced nationwide as soon as possible. Due to the inadequacy of the medical records of the emergency medical teams to record the use of automated external defibrillators by a bystander to an incident, changes to these documents should be pursued. Based on such a small cohort, it is not possible to conclude that the return of spontaneous blood circulation is correlated with the use of AEDs and public access to defibrillation PADs.

## 1. Introduction

Among all of the causes of death recorded in Europe and the world, cardiovascular diseases are a major concern because they have a high death rate [1,2]. The rate of economic development, industrialization, and population migration have begun to significantly influence the death rate from out-of-hospital cardiac arrest. By Olson et al. [3], out-of-hospital cardiac arrest accounts for more than half of all deaths from cardiovascular disease and is the first sign of heart disease in 50% of these individuals. The issue of cardiac arrest is the subject of continuous research. According to Chugh et al. [4], it should be considered a common and undifferentiated public health problem worldwide. Sudden cardiac arrest can occur anywhere people are present. Focusing only on out-of-hospital cardiac arrest, Engdahl et al. [5] concluded that OHCA most often occurs in the place of living. Other conclusions were drawn by Alqahtani et al. [1], adding public places and workplaces. Any sudden cardiac arrest requires immediate resuscitation efforts, that is, a combination of chest compressions and assisted ventilation. In addition to cardiopulmonary resuscitation (CPR), guidelines from the European Resuscitation Council (ERC) and the International Liaison Committee on Resuscitation (ILCOR) indicate that it is very important to perform defibrillation as soon as possible. Each minute of delay in performing defibrillation reduces the probability of survival after cardiac arrest by 10% [2,6,7]. Defibrillation involves the passage of a pulse of electrical energy of sufficient voltage, intensity, and power through the myocardium to invert the normal heart rhythm. Defibrillation can only be performed for 2 of the 4 mechanisms of cardiac arrest, i.e., ventricular fibrillation (VF) or ventricular tachycardia pulseless (VT). Rapid defibrillation performed within 3–5 min of cardiac arrest can ensure 50–70% survival [6,7]. It should be mentioned that defibrillation is one of the elements of the “chain of survival”, that is, actions that increase the survival rate of the cardiac arrest patient [8,9]. Presenting public access to defibrillation in Poland, it should be remembered that this is the result of a proposed strategy to improve survival after OHCA. The initiative of the ORLEN “Gift of the Heart” Foundation and the Great Orchestra of Christmas Charity Foundation can be considered the beginning of public access to defibrillation (PAD) in Poland. In 2004, the ORLEN “Gift of the Heart” Foundation located automatic defibrillators at 11 selected petrol stations as part of the “ORLEN First Aid” program. The program was expanded until 2010. Meanwhile, in 2005, the Great Orchestra of Christmas Charity Foundation transferred 100 semiautomatic defibrillators of various profiles (the total cost of purchasing AEDs was PLN 391,204.13). A total of 198 AED defibrillators were purchased on the initiative of the Great Orchestra of Christmas Charity Foundation. There are many examples of concrete cities that have more or less expanded and are expanding public access to AEDs in Poland. The city of Bydgoszcz, as part of its emergency administration and national defense tasks, maintains an AED registry, on the basis of which a map of AED locations is created. The City of Trzebinia has developed the “Trzebinia City of Safe Hearts” project. Thanks to the funds raised, among others, from a foundation from the United States (amounting to $31,400) and Polish partners and sponsors, training was conducted for residents on sudden cardiac arrest and the use of AED defibrillators, and 20 automatic defibrillators were bought. Not to be left out is the Krakow AED Network “Impulse of Life”, which has been implemented by the Municipality of Krakow in cooperation with the Krakow Ambulance Service since 2007, as part of which 31 AED defibrillators have been purchased and deployed at various locations in the city, including the Regional Bus Station, St. Mary’s Basilica, the Social Insurance Institution, the Krakow–Balice International Airport, and the John Paul II Center. The presented status of public access to defibrillation is only a brief overview of initiatives to increase safety in Poland. In order to obtain beneficial results, health education on CPR and AED use should be continuously conducted and the PAD system should be expanded [9,10,11,12,13,14,15].

The aim of this study was to analyze the use of an automated external defibrillator during the resuscitation of an adult in public places in Poland between 2015 and 2020.

## 2. Materials and Methods

The study was a retrospective research. The analyzed material included the use of AEDs in adults in the period from 1 January 2015 to 31 December 2020 in Poland. The analysis included only cases of AED use in a public place, other than a health care center, in addition to the exclusion of rescue services, i.e., the State Fire Department and the Volunteer Fire Department, which have an AED as part of their rescue equipment. The documentation obtained was analyzed, i.e., the documentation received from AED distributors (the case description data and electrocardiographic recordings from the internal memory of the AED device), and the medical documentation received from the public safety answering point and emergency medical teams. AED distributors provided data on 100 cases of AED use in a public place. The data included the date and time of AED use and the place of AED use (detailed location). In addition, 33 cases were detailed with data from the internal memory of the AED device (full record of AED operation). From the medical documentation, only the particular OHCA case was confirmed, and it was verified whether any record of AED use was made. During the research process, 100 cases of AED use during CPR performed by a bystander in a public place were confirmed. The data obtained included the date and places of AED use. In 33 cases (33%), detailed data were obtained on the course of resuscitation with an AED (a record from the AED’s internal memory representing a printout of the electrocardiogram). These data were analyzed in statistical details. The following variables were considered for analysis: the time from OHCA to AED use, the time from AED activation to the electrode activation, the number of rhythm analyses in the AED, the type of heart rhythm analyzed, and the total time of AED operation at the scene. 

The tested statistics variables were prepared in Microsoft Office Excel spreadsheet system for Windows. Statistical analysis was performed using R 3.5.3 software, with which basic descriptive statistics were analyzed. 

## 3. Results

During the analysis period (2015–2020), there were 100 recorded and documented cases of AED use in OHCA; 70% of the cases involved defibrillation. Based on 33 thoroughly documented cases of AED use, the average time elapsed from the onset of OHCA to AED use was 3 min 22 s (221.73 s). The maximum time elapsed from the onset of OHCA to the use of the AED was 10 min. Based on the statistics of the studied variable, most of the results were less than positive skewness, i.e., less than 3 min 22 s, indicating the presence of many outliers from the mean, kurtosis +2. Detailed results are shown in Table 1.

In 26 cases, the first OHCA mechanism analyzed was ventricular fibrillation (VF), accounting for 78.78%. In one case, ventricular tachycardia without pulse (VT) occurred. Non-shockable rhythms were diagnosed for a total of 6 cases: asystole(AS) *n* = 4, 12.12%; pulseless electrical activity (PEA) *n* = 2, 6.06%. Defibrillation after the first analysis was performed in 24 cases (72.73%). The time elapsed from the end of the rhythm analysis to the electrical impulse release as 7 s on average (SD = 1 s)

After 2 min (the second analysis), the AED defibrillator performed another heart rhythm analysis (according to ERC resuscitation guidelines). In two cases, no follow-up recordings were made from the AED because the patient was transferred to medical services. In the medical emergency card, the identified heart rhythm from the manual defibrillator recording was indicative of continuing VF. Based on data from 31 ECG recordings, the following was noted: VF *n* = 18; 58.06% of cases, AS *n* = 2; 6.45%, PEA *n* = 2; 6.45%. Sinus rhythm (SR) was successfully restored in 9 cases (29.03%). Defibrillation was performed in every VF case analyzed.

After another 2 min (the third analysis), the AED performed a heart rhythm analysis. In nine cases, the recording from the AED was discontinued as the patient was transferred to emergency medical services. According to the medical emergency card, the identified heart rhythm from the manual defibrillator recording indicated sustained VF—*n* = 7 or AS—*n* = 2. Based on the data from the 14 recordings, the following phenomena were identified: VF *n* = 6; 42.86% of cases, AS *n* = 4; 28.57%, PEA *n* = 1; 7.14%. Sinus rhythm (SR) was successfully restored in three cases (21.43%). Defibrillation was performed in every VF case analyzed.

After 2 min (the fourth analysis), the AED defibrillator performed another heart rhythm analysis. Only five cases recorded such a long time of use of the automated external defibrillator, based on data from five recorded cases: VF—*n* = 2; AS—*n* = 3. The percentage presentation is not justified. In three cases (21.43%), the sinus rhythm (SR) was successfully restored. Defibrillation was performed in every VF case analyzed. The mean total operating time of the AED at the scene was 8 min 5 s (M = 485.12 s), with a standard deviation of 3 min 20 s (SD = 200.15 s). The minimum AED operating time was 1 min 42 s (Min. = 102 s), and the maximum was 14 min 55 s (Max. = 895 s). The median for the parameter under study was 7 min 35 s (Me = 455 s). Descriptive statistics of the studied variable indicate that most of the results were less than the mean (positive skewness), i.e., less than 7 min 35 s, indicating the presence of many values close to the mean (kurtosis = 0—normal distribution). Table 2 shows the detailed results. 

The number of defibrillations performed with an AED at the incident location, the following results were obtained: the arithmetic mean is 1.51 (SD = 1.16), the median is 1, the minimum = 0, and the maximum = 4. The descriptive statistics of the studied variable state that most of the results are smaller than the mean (positive skewness), and the value of the feature is less concentrated with more negative kurtosis. Table 3 shows the detailed results.

## 4. Discussion

The AED is a very simple device to use. Due to its construction and functionality, it can easily be used immediately, even without any training. Once delivered to the incident location, the AED should be quickly turned on, and the operator should follow the instructions for operating the device. The voice prompts issued should be simple and correctly audible. This is guaranteed by the producer. Once the AED is turned on, the adhesive electrodes should be applied to the patient’s exposed chest. From the analyzed data, the average time for sticking the electrodes was 35.36 s (+/−31.17 s). The longest recorded time was 1 min 55 sec. Such a long time is surprising since the time for sticking electrodes is at most 10–15 s. It can be presumed that there were some obstacles, such as a wet chest, a lot of clothes, or hair on the chest.

In summary, an average of 2.79 cardiac rhythm analyses were performed during CPR (median = 2 analyses). In one case, six heart rhythm analyses were performed, and the waiting time for the arrival of emergency medical services was the longest. In 26 cases, the first OHCA mechanism analyzed was ventricular fibrillation (VF), which represented 78.78%. In one case, there was ventricular tachycardia pulseless (VT). Defibrillation after analysis was performed in 24 cases of VF (72.73%). Non-shockable rhythms (asystole, PEA) were identified for a total of eight cases. The time from the end of the rhythm analysis to the release of the electrical impulse averaged 7 s (SD = 1 s), which is confirmed by the device specifications. Each AED discharges an electrical pulse when charged to the appropriate current value. AED manufacturers state that devices can trigger an electrical impulse after 7–8 s. Summarizing the CPR, the mean total operating time of an AED at the incident location was 8 min 5 s (+/−3 min 20 s). The minimum AED operating time was 1 min 42 s, and the maximum was 14 min 55 s. Analyzing the number of defibrillations performed with the AED in the cases presented, the mean was 1.51, and the maximum number of defibrillations was 4. 

The aim of resuscitation interventions is to obtain ROSC. As is well known, rapid defibrillation is an important adjunct to chest compression and ventilation. Based on such a small cohort, it is not possible to conclude that the return of spontaneous circulation is correlated with the use of AEDs and PADs. As reported by Dwyer and Dennett [16], the use of AEDs in patients after OHCA is not related to improved survival. It should be added that the survival of patients after OHCA is also affected by the speed of arrival of emergency services. Under the Law on State Emergency Medical Services (Poland), “Art. 40. 1. Medical operations begin when the medical dispatcher receives a report or notification of the event. Upon arrival at the scene, the medical rescue team shall immediately initiate medical rescue operations” [17]. In a study by Zijlstra et al. [18], a witness to an incident used an AED an average of 2 min 39 s before the arrival of emergency medical services, and for 10.5%, the discharge occurred less than 6 min after the emergency call. Hallstrom et al. [19] noted that the average time from the call to the first rhythm analysis by paramedics was almost 3 min. The use of AEDs in Poland and around the world should be an issue subject to ongoing scientific research. Systemic programs should be implemented to expand public access to defibrillation. An important initiative for national policy makers should be the creation of a national registry of AED defibrillators and a system for reporting their use. It is worth being open to the new technological advances. For example, drones equipped with AEDs should be used more often, as this can improve the survival rate of patients after SCA. A recommendation to include information on AED use in paramedics’ medical charts should be considered. The ERC Resuscitation Guidelines 2021, published in 2021, single out a topic dedicated to CPR education and implementation among the population. Systems saving lives addressed to government, health, and education system managers; health care professionals; educators; students; and the population gives guidance on how to improve the management of cardiac arrest patients. Based on evidence-based medicine, guidance is provided on interventions to improve the survival rates of patients after OHCA, among other things. The implementation of a system of CPR and AED training, conducting research on CPR and AED use, should aim to increase the percentage of people undertaking CPR and using AEDs. The emergency medical system should also include the creation of a database of public defibrillators (PADs) in its call-handling system and use it to direct the witness to an incident to the nearest PAD. The role of the medical dispatcher should be highly emphasized in this topic. It should be made mandatory to register AEDs that are owned. The importance of new technologies, i.e., smartphone apps, should be strengthened. These can be used by the public to locate public AEDs and to record the location of public AEDs that are not yet displayed on the smartphone app. Cell phone technologies can also be used to alert citizens about OHCA. These have already been implemented in many countries. Alerting citizens about OHCA patients is associated with a higher rate of bystander initiated defibrillation, AED use before ambulance arrival, and survival at hospital discharge or after 30 days. In Poland, such a system has not yet been developed and implemented. Only AED maps are available online and in the form of a smartphone app (Save with Heart—AED map; Save a Life; and Staying Alive). In early 2022, thanks to the efforts of the OpenStreetMap Poland (OSM) Association, a website was launched that displays all of the defibrillator (AED) locations in Poland. This registry can be easily updated by any user with an account on OSM [20,21,22,23,24,25]. 

## 5. Conclusions

The study shows that 100 cases of use of an AED placed in a public place in Poland were recorded between 2015 and 2020. This result could be higher, but the study’s methodology and limited access to data only allowed for this result. Based on such a small cohort, it is not possible to conclude that the return of spontaneous blood circulation is correlated with the use of AEDs and PADs. In Poland, there are no legal acts on the registration of AED and their implementation. Appropriate registries should be introduced nationwide as soon as possible. Due to the inadequacy of the medical records of emergency medical teams to record the use of automated external defibrillators by a bystander to an incident, changes to these documents should be pursued. 

## Figures and Tables

**Table 1:** Basic descriptive statistics for a number of defibrillations and several rhythm analyses by AED (*n* = 33).

	M	Me	Min.	Max.	SD	Sk.	Kurt.
Occurrence of OHCA to use AED time in seconds [s]	221.73 (3 min 22 s)	180.00 (3 min)	60.00 (1 min)	600.00 (10 min)	125.98 (2 min 6 s)	1.53	2.07
Occurrence from AED activation to electrode application time in seconds [s]	35.36	22.00	4.00	115.00 (1 min 55 s)	31.17	1.05	0.34
Number of AED rhythm analyses	2.79	2.00	1.00	6.00	1.17	0.94	0.60

Explanation of abbreviations used: M—arithmetic mean; Me—median; Min.—minimum; Max.—maximum; SD—standard deviation; Sk.—skewness; Kurt.—kurtosis; AED—automated external defibrillator; OHCA—out-of-hospital sudden cardiac arrest; s—seconds, and min—minutes.

**Table 2:** Baseline statistics for AED use time (*n* = 33).

	M	Me	Min.	Max.	SD	Sk.	Kurt.
AED operation time [s]	485.12 (8 min 5 s)	455.00 (7 min 35 s)	102.00 (1 min 42 s)	895.00 (14 min 55 s)	200.15 (3 min 20 s)	0.35	0.02

Explanation of abbreviations used: M—arithmetic mean; Me—median; Min.—minimum; Max.—maximum; SD—standard deviation; Sk—skewness; Kurt.—kurtosis; AED—automated external defibrillator; and s—seconds.

**Table 3:** Basic statistics on the number of defibrillations done with AED (*n* = 33).

	M	Me	Min.	Max.	SD	Sk.	Kurt.
Number of AED defibrillations	1.51	1.00	0.00	4.00	1.16	0.34	−0.78

Explanation of abbreviations used: M—arithmetic mean; Me—median; Min.—minimum; Max.—maximum; SD—standard deviation; Sk.—skewness; Kurt.—kurtosis; AED—automated external defibrillator; and s—seconds.

## Data Availability

The data presented in this study are available on request from the corresponding author.
